# Cone beam computed tomography imaging of sagittal positions of the mandibular prominence and maxillary central incisors in adult Chinese Han men as an aesthetic profile determinant

**DOI:** 10.1097/MD.0000000000022778

**Published:** 2020-10-23

**Authors:** Pei Miao, Jie Gao, Zhiyao Lu, Zuolin Jin

**Affiliations:** a State Key Laboratory of Military Stomatology & National Clinical Research Center for Oral Diseases & Shaanxi Clinical Research Center for Oral Diseases, Department of Orthodontics, School of Stomatology, The Fourth Military Medical University; b Department of Stomatology, Xi’an First Hospital, Xi’an, Shaanxi, China.

**Keywords:** aesthetic profile, Andrews the 6 elements of orofacial harmony, cephalometry, mandibular prominence, maxillary central incisors

## Abstract

**Background:**

To analyze the sagittal positions of the mandibular prominence and maxillary central incisors in adult Chinese Han men to establish their aesthetic profile characteristics.

**Methods:**

Seventy-four Chinese Han men aged 18 to 40 years underwent cone beam computed tomography for detecting the distances between Glabella and Subnasale, Subnasale and Menthon of soft tissue, Condyle and Gonion, Pogonion and Pogonion's Anterior Limit Line, Facial Axis point of maxillary central incisor and the Goal Anterior Limit Line as well as the angle of the Occlusal Plane. Dolphin Imaging and Photoshop software packages were used to generate silhouette profiles. Thirteen orthodontists assessed the silhouette profiles and assigned visual analog scale scores. Scores >70 were assigned to the aesthetic (group 1), scores of 60to 70 to the general (group 2), scores of 50 to 60 to the acceptable (group 3), and scores of <50 to the unaesthetic profile (group 4).

**Results:**

A total of 15 men were assigned to group 1, 35 to group 2, 14 to group 3, and 10 to group 4. There were no significant differences in the variables examined between groups 1, 2, and 3, but comparing group 1 with group 4, Pogonion and Pogonion's Anterior Limit Line (1.16 ± 2.61 mm vs −1.44 ± 2.92 mm, *P* = .046) and Facial Axis-Goal Anterior Limit Line (−0.61 ± 2.54 mm vs 1.70 ± 2.62 mm, *P* = .038) there were significant differences.

**Conclusion:**

Compared with the unaesthetic profile group, the sagittal positions of the maxillary central incisors were slightly posterior, and the chin was slightly anterior in adult Chinese Han men with an aesthetic profile.

## 1 Introduction

Over the years, the focus on orthodontic treatments has changed from teeth occlusions to the harmonious and esthetic soft tissue. In 1991, Andrews proposed the 6 Elements of Orofacial Harmony that combined external soft tissue points and X-ray cephalometry. The 6 elements comprised: I the arch; II the Anteroposterior (AP) jaw positions; III jaw width; IV jaw height; V chin prominence; and VI occlusion.^[1]^ Especially, the protrusion and retraction of the mandible greatly affects the facial profile, which is often a major factor for patients seeking orthodontic treatment and surgery^[2]^ and the forehead has been described as an important landmark for AP maxillary incisor positioning for improving facial harmony.^[3]^ Also in another study, protrusive mandibles in males and females were judged to be the least attractive in a study of Chinese perspectives on facial profile attractiveness.^[4]^ Comparing esthetic perceptions of laypersons, dental students, general practitioners, oral surgeons and orthodontists, apart from laypersons, all other groups cited the chin as the most influential part for determining facial profile attractiveness, with bimaxillary protrusion and mandibular retrusion being the least attractive profiles in both males and females.^[5]^

Orthodontic treatment plans require reference cephalometric standards across different ages and genders. However, ethnic differences in facial features may also create different aesthetic standards between Chinese and Western individuals.^[6]^

Here, we used 3 of Andrews’ 6 Elements of Orofacial Harmony as a reference index to analyze the sagittal positions of the mandibular prominence and maxillary central incisors of adult Chinese Han men to provide reference indicators for esthetic perception.

## 2 Methods

### 2.1 Sample selection

In this comparative, prospective study, 74 Chinese Han men aged 18 to 40 years with symmetrical maxillofacial features, complete and permanent dentition with a dentition crowding degree <1 mm, a normal overbite and overjet, a class I skeletal pattern, and a class I occlusion were recruited. There were no histories of maxillofacial trauma, surgery, orthodontic treatment or temporomandibular joint disorder. The ethical committee of the Stomatological Hospital of The Fourth Military Medical University approved the study (Ref: IRB-REV-2015001).

### 2.2 Experimental methods

#### 2.2.1 Generation of silhouette profiles

**Figure 1:**
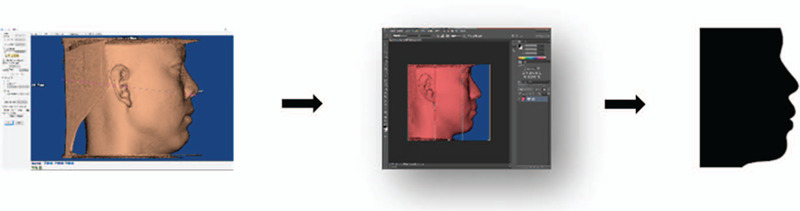
Generation of silhouette profiles using Dolphin imaging and Photoshop software.

Each patient was positioned in an upright position, with the head also relaxed in an upright position, lips closed and eyes looking straight ahead. The teeth were maintained in the intercuspal position. To avoid the deformation of chin soft tissue under pressure, the chin stent was removed during KaVo 3D eXam cone-beam computed tomography (CBCT) (KaKo Kerr, Orange, CA). The CBCT data were saved as DICOM files, which were then imported into the Dolphin Imaging 11.8 software package to reconstruct the 3-dimensional soft tissue profiles. Silhouette profiles were generated using Photoshop software (Adobe, San Jose, CA) (Fig. 1).

Primary outcome was the sagittal positions of the mandibular prominence and maxillary central incisors of adult Chinese Han men measured by Pogonion (Po) and Pogonion's Anterior Limit Line (PALL) (PO-PALL, mm) and Facial Axis (FA) point of maxillary central incisor and the Goal Anterior Limit Line (GALL) (FA-GALL, mm).

Secondary outcomes were the mean and standard deviations of all indicators of all enrolled subjects (n = 74) for esthetic perception including elements II, IV, and V of the Andrews’ 6 Elements of Orofacial Harmony. The variables were the distances between the Glabella (G) and Subnasale (Sn) (G-Sn), Sn and Menthon of soft tissue (Me’) (Sn-Me’), Condyle (Co) and Gonion (Go) (Co-Go), PO-PALL, FA-GALL as well as the angle of the occlusal plane (OP).

#### 2.2.2 Visual analog scale (VAS) scoring

Thirteen orthodontists with more than 3 years’ experience in the Orthodontics Department of the Stomatological Hospital of The Fourth Military Medical University served as the evaluators. Each evaluator was required to score the black-and-white facial silhouette profiles of 74 upright-sitting men. Each silhouette profile was judged by each evaluator using a 100-point VAS score to express the degree of attraction. VAS scores of 70 to 100 represented aesthetic profiles (group 1), scores of 60 to 70 general profiles (group 2), scores of 50 to 60 acceptable profiles, (group 3), and 0 to 50 unaesthetic profiles (group 4).^[4,7]^ The silhouette profiles were scored by each orthodontist once a week for 3 consecutive weeks and then the scores were averaged.

#### 2.2.3 Andrews’ 6 elements of orofacial harmony measurements

**Figure 2:**
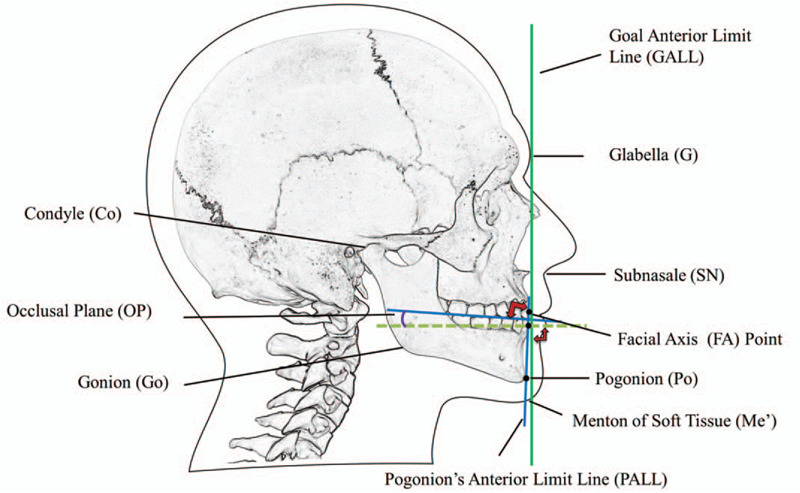
Scheme of the measurement points and axes used in the present study.

The elements II, IV, and V of Andrews’ 6 Elements of Orofacial Harmony were measured with Dolphin Imaging 11.8 software (Dolphin Imaging & Management Solutions, Chatsworth, CA). The variables were the distances between the G-Sn, Sn-Me’, Co-Go, PO-PALL, FA-GALL as well as the angle of the OP (Fig. 2). Since in most of Chinese Han men the Goal Anterior Limit Line passes through the glabella,^[8]^ the forehead's facial axis point was replaced by the glabella when measuring the distance between the maxillary central incisor’ FA point and GALL.

Finally, the results of the VAS scoring were compared with the data collected using Dolphin Imaging software.

### 2.3 Statistical methods

The results are expressed as means ± standard deviation. An independent sample *t*-test was used for comparisons between the groups. If the normality test revealed that the data was not normally distributed, a non-parametric rank sum test (Mann–Whitney) was employed. *P*-values < .05 were considered to be statistically significant.

## 3 Results

### 3.1 Subjects aesthetic grouping and face indices measurements

**Figure 3:**
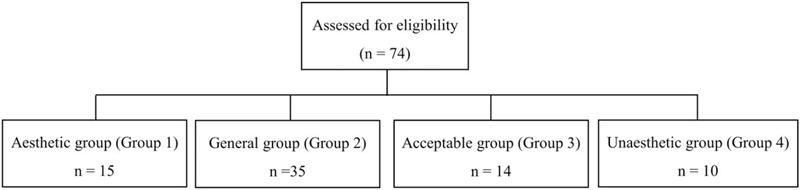
Flowchart of the present study.

The men were divided into 4 groups according to the VAS score as follows: 15 men were assigned to group 1, 35 to group 2, 14 to group 3, and 10 to the group 4 (Fig. 3). The overall results of the indicated variables in adult Chinese Han men are show in Table 1.

**Table 1:**
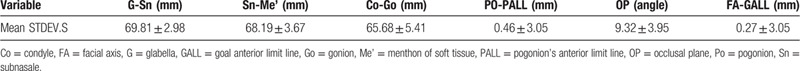
Face indices measurements of adult Chinese Han men (n = 74).

### 3.2 Outcomes

The results of comparisons between 2 groups are listed in Tables 2–4. The primary outcome indicators PO-PALL and FA-GALL were significant different between group 1 and group 4 (*P* = .046 and *P* = .038) suggesting that these 2 indices might be aesthetic indicators. For the secondary outcomes we found that in the aesthetic group, the G-Sn distance was 69.04 ± 3.58 mm, the Sn-Me distance was 68.31 ± 2.8 mm, the Co-Go distance was 66.37 ± 4.49 mm, the Po-PALL distance was 1.16 ± 2.61 mm, the FA-GALL distance was −0.61 ± 2.54 mm and the OP angle was 9.77° ± 4.10°. However, there were no statistical differences in the elements II, IV, and V of the Andrews’ 6 Elements of Orofacial Harmony between the men in the aesthetic group 1 and those in the general group 2, as well as between men in aesthetic group 1 and those in the acceptable group 3. In addition, other indicators than PO-PALL and FA-GALL were also not significantly different between group 1 and group 4. Table 5 shows the 95% confidence intervals for the mean values of all indicators comparing all other participants with group 1.

**Table 2:**
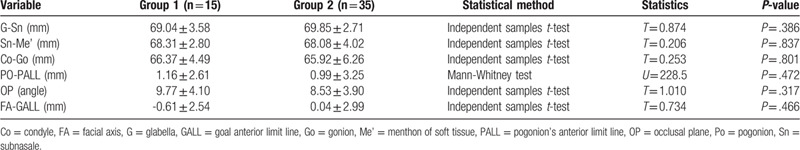
Comparison of face indices between group 1 and group 2.

**Table 3:**
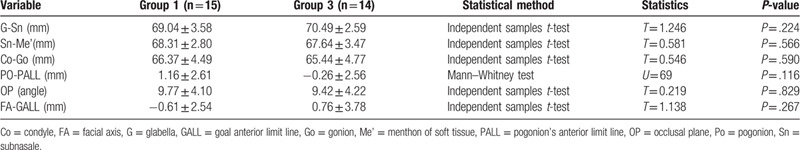
Comparison of face indices between group 1 and group 3.

**Table 4:**
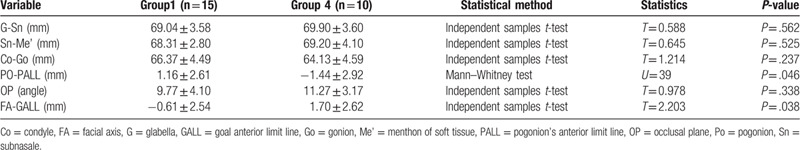
Comparison of face indices between group 1 and group 4.

**Table 5:**
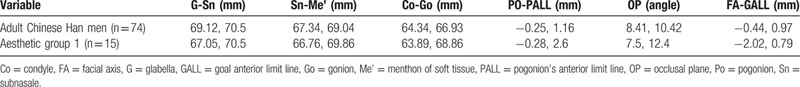
95% Confidence intervals for orofacial harmony measurements of adult Chinese Han men.

## 4 Discussion

In the orthodontic assessment, the conventional clinical detection methods are mainly panoramic radiograph, X-ray cephalometric imaging, periapical film, and model, thus the CBCT data were used to reconstruct the 3D soft tissue profiles instead of 2D images. Because the influence of magnification and overlap from 2D images, the positioning, and measurement of some anatomic structures lack accuracy, resulting in large differences in measurement values and poor repeatability. However, CBCT can display the craniofacial reconstruction of the 3D soft tissue profiles without overlapping distortion, and the tissue cross-section provided by CBCT can accurately locate the anatomical markers required for cephalometric analyses. Therefore, CBCT can be used to reconstruct the measured values of images with high accuracy. Through proprietary software, physicians can also overlap the images before and after treatment, and make an animation to see the changes in teeth, craniofacial and soft tissue, which is of great help in evaluating the effects of orthodontic treatment and changes after orthodontic surgery.

Soh et al^[4]^ concluded that the profiles of Chinese men with mildly protruding upper lips were more attractive than those of Chinese men with bimaxillary retrusion with flatter lips. Andrews proposed that the optimal position of the FA point of maxillary central incisor should be on GALL. We found that the mean distance between the FA point and GALL was 0.27 ± 3.05 mm for adult Chinese Han men, and the 95% confidence interval was −0.44 to 0.97 mm. For men with aesthetic profiles, the mean distance between the FA point and GALL lines was −0.61 ± 2.54 mm and the 95% confidence interval was −2.02 to 0.79 mm. The maxillary central incisor's FA point of adult Chinese Han men with aesthetic profiles was more posterior to the GALL lines. In a comparison of FA to GALL data from aesthetic and unaesthetic groups, Chinese individuals favored flat or slightly retracted maxillary profiles and disliked protruded maxillary profiles. This finding may be related to our decision to define the GALL through the glabella or the Chinese globalization, which might have influenced the aesthetic judgment, as Chinese might favor a flat profile with slight retraction.

Johnston et al^[9]^ used silhouette profiles to investigate the relationship between mandibular prominence and attractiveness, and he proposed an Eastman normal SNB angle, representing the relative AP position of the mandible to the cranial base of 78° as the most attractive. The use of the SNB angle to estimate the attractiveness of individuals with mandibular prominence does not always take into account variations in the SNB angle with the sella/nasion plane. However, it had no effect on the shape of the lower lip and the sagittal position of pogonion of soft tissue. Naini et al^[2]^ reported that slight mandibular retraction or protrusion was considered to be more attractive, but attractiveness decreased with increased mandibular retraction or protrusion. These authors also indicated that the most attractive profile of the mandibular soft tissue was the one where the pogonion of soft tissue was located on the plumb line of the sub-nasal point and the lower lip was slightly behind the upper lip. In other high-scoring profiles, the pogonion of soft tissue was located between 4 mm behind and 2 mm ahead of the plumb line.

Andrews proposed that the AP prominence of the hard chin was optimal, when the Po point was equal in prominence to the PALL. We found that the mean distance between the Po point and PALL was 0.46 ± 3.05 mm for adult Chinese Han men, and the 95% confidence interval was −0.25 to 1.16 mm. For adult Chinese Han men with aesthetic profiles, the mean distance between the Po point and PALL was 1.16 ± 2.61 mm, and the 95% confidence interval was −0.28 to 2.6 mm. The Po point of men with aesthetic profiles was anterior to the PALL line. A comparison of Po to PALL data from aesthetic and unaesthetic groups revealed that Chinese men favored slightly protruding mandibular profiles and disliked retracting mandibular profiles.

No significant differences (*P* > .05) in G-Sn, Sn-Me’, Co-Go, and OP were observed among the 4 groups. For adult Chinese Han men with individual normal occlusions, G-Sn, Sn-Me’, Co-Go, and OP were not the key indicators for judging attractiveness according to Andrews’ 6 Elements of Orofacial Harmony.

One limitation of the present study was the relatively small sample sizes of the subgroups that might not represent the general population of Chinese men and the generalization needs further studies with larger cohort numbers.

## 5 Conclusion

Compared with the unaesthetic population, for the aesthetic population the sagittal positions of the maxillary central incisors were slightly posterior, which means that the nasolabial angle was slightly greater and the upper lip was relatively flat, while the mandibular prominence ensured that the mentolabial angle was slightly smaller and the chin slightly more prominent. The Chinese favor a flat profile like a Caucasian rather than a convex profile like a Mongolian.

## Author contributions

**Conceptualization:** Pei Miao, Zuolin Jin.

**Data curation:** Pei Miao, Jie Gao, Zhiyao Lu.

**Formal analysis:** Pei Miao, Zuolin Jin.

**Investigation:** Zuolin Jin.

**Supervision:** Zuolin Jin.

**Validation:** Pei Miao, Zuolin Jin.

**Writing – original draft:** Pei Miao.
